# Two Sports, Two Systems, One Goal: A Comparative Study of Concussion Policies and Practices of the Australian Football League and Hockey Canada

**DOI:** 10.3389/fspor.2021.672895

**Published:** 2021-07-06

**Authors:** Annette Greenhow, Alison Doherty

**Affiliations:** ^1^Faculty of Law, Bond University, Gold Coast, QLD, Australia; ^2^School of Kinesiology, Western University, London, ON, Canada

**Keywords:** concussion, brain injury, sport governance, regulation, national sporting organisation, Australian Football League, Hockey Canada

## Abstract

Concussion in sport is today regarded as both a public health issue and high profile injury concern in many contact and collision sports. This paper undertakes a comparative review of the current policies and practices of two high profile national sporting organisations of such sports—the Australian Football League (AFL) and Hockey Canada (HC)—in governing the issue as a regulatory concern. By examining the policies and practices of the AFL and HC, this study aims to identify common themes, divergent practices, and nuanced sport-specific approaches to develop understandings on the regulation and governance of this high profile sports injury. The paper aims to contribute to understanding concussion as a regulatory concern, while at the same time recognising the heterogeneity of sport and reinforcing nuanced understandings that align to specific social and cultural settings. We make recommendations based on regulatory and cultural legitimacy. The paper concludes that these NSOs are institutional actors with historical and cultural roots who assert regulatory legitimacy by steering and influencing behaviour and directing the regulatory agenda to manage and mitigate the harm associated with concussion.

## Introduction

This paper examines the regulation of concussion in the context of sport governance. Our focus is on the role of the National Sporting Organisation (NSO) as the institutional actor steering and influencing behaviour by setting and directing the regulatory agenda. The paper contributes to understanding the regulatory role of NSOs in responding to concussion as a concern within their sport, recognising the heterogeneity of sport, and nuanced understandings that align to specific social and cultural settings.

We acknowledge that other actors participate in this regulatory space. However, in the context of this paper, our goal is to identify and compare the policies, regulations and guidelines as regulatory mechanisms employed by the NSO to achieve the objective of managing and mitigating concussion within the sport. Thus, our research question is what are the similarities, differences, and nuances in the way the AFL and HC, as institutional actors, govern through regulation to manage and minimise the harm associated with concussion?

To address this question, the paper proceeds as follows. First, we establish the foundations of the study by identifying relevant theories, conceptualising key terms and briefly framing the public and private dimensions of sport-related concussion. Next, we examine the specific governance characteristics of the AFL and HC as regulatory actors for their sport. As this paper focusses on concussion in sport as a regulatable concern, we then turn to consider the specific policies, regulations and guidelines deployed by each NSO to address the issue within their sport. Specifically, we examine responses across four areas: concussion prevention; concussion injury management; concussion education and advocacy, and concussion research. Our aim is to identify common themes, divergent practices, and nuanced sport-specific approaches. We then propose several recommendations and identify areas for future research consideration including a larger scale comparative study evaluating the effectiveness of NSOs' concussion strategies.

As this paper involves a comparative analysis of the regulatory arrangements across two sports, we adopted a qualitative research methodology to understand the similarities, differences and nuances in the way institutional actors govern within their respective regulatory environments. We focus on the policies and practices that address the phenomena of interest (Peters and Fontaine, 2020), relying on documentary analysis of public records available to February 2021 as evidence of those policies and practices. We sought and examined a range of materials, obtained from a variety of written sources, that inform our understanding of the focus and engagement of the NSOs in the realm of sport-related concussion. This included a thorough screening of their respective websites for insights to governance, regulation and organisational processes, and concussion-related statements and resources. We also collected relevant materials from searches beyond the NSOs themselves, including media reports and various stakeholder organisations and initiatives connected with the NSOs' concussion efforts. In our search and examination of materials, we were guided by the four categories of concussion prevention, concussion injury management, concussion education and concussion research. The materials were read and examined for information that would help address the similarities, differences, and nuances in the way the AFL and HC, as institutional actors, govern through regulation to manage and minimise the harm associated with concussion. Our analysis of secondary data provides a cogent foundation for understanding the regulatory legitimacy of these NSOs over sport-related concussion as a regulatable concern.

In selecting the AFL and HC as cases to review, we adopted what Peters describes as a “most-similar systems” design; by selecting cases that are as similar as possible but vary on some key feature (Peters and Fontaine, 2020). The AFL and HC are both NSOs, each representing high profile contact/collision sports within their respective jurisdictions but, as this paper explains, with different systems of governance and in different social and cultural settings. This paper also explains that the sports of Australian football and ice hockey each involve bodily contact with exposure to the risks of concussive and sub-concussive injuries. Therefore, we justify the selection of the AFL and HC as NSOs representing two sports operating under two governance systems but with a common goal of managing and mitigating the harm associated with concussion.

It falls beyond the scope of this paper to evaluate the effectiveness of the NSOs' regulatory mechanisms. Further, we do not undertake a comparative analysis of all micro- and macro-level drivers that influence how NSOs steer policies to address concussion. Consequently, this paper does not represent an exhaustive examination of every aspect of the regulatory and governance processes employed by the AFL and HC. Further, the paper does not compare legal or political systems within which each NSO operates nor do we expand into international and transnational sport macro-level governance arrangements.

We draw from our disciplinary backgrounds in law, regulation, governance and sport management to understand concussion as a sports phenomenon. Our disciplinary perspectives enable us to apply different theoretical lenses. We turn now to a consideration of these lenses.

## Theoretical Lenses, Key Terms, and Problem Identification

### Theories of Regulation

In his comprehensive analysis, Windholz (2018) categorises regulatory theories into four groups: public interest theories, private interest theories, institutional theories, and ideational theories. These theories “justify and explain” regulation as a distinct and prominent form of governance (Windholz, 2018). Regulation is itself recognised as an “inter-disciplinary melting pot,” and the application of regulatory theories “produces a richer environment from which to examine and analyse the regulation endeavor” (Windholz, 2018, p. 13).

#### Regulation

Scholars have not settled on a universally accepted definition of regulation (Baldwin et al., 2011; Freiberg, 2017; Koop and Lodge, 2017; Windholz, 2018). According to Hancher and Moran (1989), regulation is the defining feature of any system of social organisation but the task of identifying what regulation is “obscure[s] as much as it illuminates” (p. 271). Despite observations that regulation means “different things to different people” (Levi-Faur, 2012), behaviour modification is a common theme (Black, 2002; Koop and Lodge, 2017; Windholz, 2018).

This paper is informed by Black's description of regulation as:

the sustained and focused attempt to alter the behaviour of others according to defined standards and purposes with the intention of producing a broadly identified outcome or outcomes, which *may* [emphasis added] involve mechanisms of standard-setting, information-gathering and behaviour modification (Black, 2002, p. 26).

Black's pluralistic view of regulation is suitable for use in the context of sport due to many deeply entrenched cultural and customary assumptions about the legitimacy of actors and arrangements within this regulatory arena.

#### Regulation by Non-state Actors

Typically, the governance of sport is self-regulated by a network of non-state actors with little direct interference or regulatory control from government (Weatherill, 2017). Indeed, the autonomy of private non-state actors governing sport is a deeply entrenched characteristic, and often used to jealously guard and protect regulatory authority (Olympic Charter 2020, Fundamental Principles of Olympic, principle 5). Bachynski (2019a) highlights how early non-state actors from within sport relied upon deeply entrenched cultural and social beliefs to garner legitimacy in framing problems that fall within their regulatory remit. These characteristics align with sociological institutionalism and underpin claims for regulatory legitimacy by NSOs.

While much of the regulatory literature focuses on public sector governance, the theories of regulation can equally apply to non-state settings and are useful to help explain how regulation occurs and support claims of regulatory legitimacy by non-state actors. Grabosky (2013) identifies a variety of non-state actors, operating beyond state auspices, operating within the “cosmopolitan nature of contemporary regulatory space” and reflecting modern concepts of regulatory pluralism.

Regulatory actors can include individuals, peer groups, associations, firms (both incorporated and unincorporated), unions, NGOs, various levels of state and government actors, supra-governmental agencies, standard-setting organisations and global organisations (Freiberg, 2017). The emphasis is not so much on the classification of state or non-state actors who regulate or the formal or informal structures through which they operate. Instead, the emphasis is on the objective or goal of the regulatory endeavour, described by Black (2002) as the making and implementing of policies to steer and influence behaviour.

In this paper, our focus is on the NSO as the institutional actor promulgating and implementing policies. As NSOs, they assert legitimacy for these rules-making and standard setting functions with a sport. Institutional theory proffers insights into the regulatory legitimacy of institutional actors and the instruments they use to steer and influence behaviour.

According to sociological or new institutionalism, institutions are viewed as an “expression of the way the world works, embodying and embedding shared cultural understandings of what is socially acceptable and legitimate” (Windholz, 2018, p. 53). Thus, regulatory legitimacy is not based in politics but instead reflects deeply entrenched social norms, beliefs and relations “that explain and justify the proper allocation of power and resources” (Fiss, 2008, p. 391). Peters (2016, p. 310) identifies this perspective as particularly suited to the examination of micro-level governance, where “actors within a single policy area are more likely to share values, symbols and other aspects of a normative structure”—such as within an NSO—than across broader structures characterised by macro-governance (Fiss, 2008). Batuev and Robinson (2019, p. 169) further distinguish between regulatory legitimacy as founded in the “valid, objective social feature[s]” of an institution, and cultural legitimacy of an institution that is founded in the “social object as right” within its broader field. Thus, macro-level cultural legitimacy may be seen as a foundational condition of micro-level regulatory legitimacy in the sporting context (Batuev and Robinson, 2019).

Sociological institutionalism arises in settings that rely on a variety of normative instruments to exert control over or at least manage the members of the institution, recognising the influence and emphasis on values as a means of “producing governance.” In sport, where strong sociological backgrounds and entrenched cultural values exist, we consider sociological institutionalism a suitable theory to assist in describing and explaining the role of NSOs in asserting regulatory legitimacy and being recognised as socially acceptable and legitimate institutional actors with “strong credibility and acceptability” to govern. We examine this in further detail later in this paper when reviewing the nature and scope of the NSOs actions in steering and influencing behaviour in regulating concussion.

### Defining Key Terms

#### Governance

Governance has been described as the act of steering or directing but there is no universally accepted definition across multiple settings. Indeed, Ansell and Torfing (2016, p. 2) explain that “governance is a popular but notoriously slippery term.” Lawyers tend to view governance as the application of rules to govern behaviour enforced through institutions relevant to a particular area of law. Corporate governance, for example, is regarded as the institutionalised interaction among many players involved in the process of directing and controlling private firms (Ansell and Torfing, 2016).

Governance derives from the Latin word “*gubernor”* and means to “pilot, steer or direct” (Windholz, 2018). While the act of steering and directing is central to the meaning of governance, there exists “great variation with respect to what should be steered or directed, by whom, and how…” (Windholz, 2018, p. 5) Within this definition, governing is undertaken with purposive intent rather than passive acquiescence.

Windholz (2018), citing Knill and Tosun (2012), explains the three forms of governance as being hierarchical governance, governance by markets and governance through networks. We are interested in this latter mode of governance as involving collaboration by actors to achieve common goals. In this paper, we compare governance of the AFL and HC in the context of policies, regulations, and guidelines to address concussion.

In terms of activities, Peters (2016) identifies several central activities that shape and define governance. These include goal selection, policy formation, resource attachment, implementation, evaluation, and feedback. We examine the first three of these activities later in the paper in description and comparison of the concussion-specific policies, regulations and guidelines of the AFL and HC.

#### Sport Governance

Of particular interest in the sport management literature are the governance models of various countries and sport codes. Amateur sport in Canada and in Australia (for the most part) follows a federal model of governance, which aligns with their national governance systems, both of which have British colonial histories (O'Boyle and Shilbury, 2016). In a federal model, sport is governed through a hierarchical but semi-autonomous system of sport organisations at the national (NSO), state (SSO, Australia) or provincial/territorial (PTSO, Canada), and community (CSO) levels. As a federated structure, similar to political governance, NSOs and SSOs/PTSOs are designated representatives of amateur sport at their respective levels and operate with some independence. Nonetheless, in both countries, an arms-length agency oversees sport for the federal government—Sport Australia and Sport Canada—and NSOs are recognised by these agencies (and thus the federal governments) as the official governing bodies and representatives of their respective sports in and for the countries (Cuskelly et al., 2013; Doherty and Clutterbuck, 2013). A key feature of a federated structure is the delegate model of board composition, which serves as a mechanism for coordination between NSOs and SSO/PTSOs. However, many NSOs have transitioned to more independent boards composed of members appointed for a skillset that is lacking in the composition of the board (O'Boyle and Shilbury, 2020).

A challenge of the federal system of sport governance is the often-overlapping power and responsibility of the NSO and SSO/PTSO in a given sport, with different strategic and operational plans that, nonetheless, address similar objectives (O'Boyle and Shilbury, 2020). This can undermine a “whole-of-sport” perspective.

Importantly, the AFL operates a dual model of governance incorporating elements of both professional league governance and amateur sport governance [Australian Football League, 2018 cl. 4 (a); MacDonald and Ramsay, 2016]. The AFL governance structure evolved with the transition from professional league organiser to later being designated as the NSO for the sport in the 1990s. The differences in these governance characteristics are discussed later in the paper.

As this paper is concerned with the governance by the NSOs as private actors who play significant regulatory roles in sport, we conceptualise governance as the structures and processes used by the NSO steering and influencing behaviour toward the goal of managing and mitigating the harm associated with concussion in their sport.

#### Regulatory Legitimacy

Regulatory legitimacy is essentially the credibility and acceptability of policy-making authority. Legitimacy is a key attribute of effective regulation or regulatory “success” (Baldwin et al., 2011; Windholz, 2018) By understanding how NSOs assert regulatory legitimacy through their policy-making actions, such as promulgating rules and guidelines to steer and influence behaviour in their field, the NSOs establish and reinforce their regulatory mandate. It is beyond the scope of this paper to evaluate measures of regulatory “success.” We simply note that the act of policy-making asserts regulatory legitimacy and is an “essential resource of policy-makers” (Windholz, 2018). As our aim in this paper is to identify how the NSOs assert regulatory legitimacy over concussion in sport, we now turn to examine the complexities associated with framing the problem as a regulatable concern.

### Framing the Problem

Determining whether a problem is selected as one for active consideration and, consequently, who should set and direct the regulatory agenda, depends on whether the problem is framed as a public or private problem. Indeed, issue definition and diagnosis are critical foundational steps in the regulatory policy lifecycle (Freiberg, 2017; Windholz, 2018).

Doherty (2012) first identified the complexities associated with concussion in sport when examining the challenges of “fitting” it neatly within disciplinary lines. She explains the problem as having been framed as medical issue, a legal issue, a social and cultural issue, and drew from different perspectives to illustrate the “different angles” in explaining concussion as a sports phenomenon.

From a governance and regulatory perspective, Malcolm explains that biomedical science alone is “insufficient” to address the problem (Malcolm, 2019). Malcolm (2019), citing Greenhow and East (2015), notes that sport-related concussion is a “wicked” problem; one that is “complex, difficult to define, evolving and has many interdependencies and many stakeholders” (p. 70).

#### Biomedical Perspectives

The biomedical sciences have laid claim to the problem as being a medical one and sports physicians with experience in sport medicine have published widely in the field (Cantu, 1986; McCrory, 2001; Patricios and Makdissi, 2014; Davis et al., 2017). Consequently, the sports medicine and biomedical scientific literature are replete with studies on the medical construction of sport-related concussion as a clinical syndrome (Aubry et al., 2002; Dashnaw et al., 2012).

Today, concussion in sport is identified as the “number one injury risk” in contact and collision sports (Raftery et al., 2016), with many sports now taking steps toward prevention, injury management, education and advocacy and research. Adopting the label as being a “sports injury” has enabled private actors, namely the NSOs, to steer and influence behaviour by asserting regulatory legitimacy over the problem as falling within their remit.

There is no universal consensus as to what precisely defines a concussion. A review of the literature identifies concussion as being labelled as a “brain injury,” a “mild brain injury,” a “head injury” or a “mild Traumatic Brain Injury” (mTBI) (McCrory, 2001; Patricios and Makdissi, 2014). For our purposes, we are guided by the latest International Consensus Statement generated by the Concussion in Sport Group (CISG) wherein scholars and biomedical clinicians define sport-related concussion *as a traumatic brain injury induced by biomechanical forces* (McCrory et al., 2017).

Another challenge associated with concussion is the latent nature of the injury, often described as an “invisible injury” (Bloom et al., 2004; Caron et al., 2013); where “vague and heterogeneous symptoms” constrain accurate and timely diagnosis (McNamee et al., 2015, p. 193). With no visible signs or symptoms as to the extent of injury, there exists no objective measure or complete diagnostic tool currently available to accurately assess when a player is asymptomatic and ready to return to activity. Mismanagement of the initial concussive injury is the problem; where a player is susceptible to more serious harm due to the brain being in an “injury induced vulnerable state” (Wojtys et al., 1999, p. 677).

Accurate injury diagnosis relies upon full and frank self-reporting by patients. Herein lies another challenge in sport. In a competitive sporting environment, performance-driven motivations, structural constraints, economic imperatives, and sociocultural influences are likely to present barriers to voluntary self-reporting by players, leading to serious concerns of under-reporting, and playing while injured (Cusimano et al., 2017). Bachynski (2019a) highlights that concussion under-reporting is a reoccurring theme across multiple sport settings, embedded in the social and cultural beliefs that participation and playing through pain are hallmark features of masculinity.

The NSOs were early actors in framing and labelling concussion in sport as a “sports injury.” However, concerns about the wider social impact necessitates a brief review of whether the problem also has public health dimensions.

#### Public Health Perspectives

As we have recently witnessed during the COVID-19 pandemic, government actors, as guardians of the public health, have significant roles to play in regulating public health concerns. Gostin and Wiley (2016) explain that the word “public” in public health has two overlapping meanings—one that explains the *entity* that takes responsibility for the public's health (“who” regulates), and the other focussed on the *beneficiaries*—those who have a legitimate expectation of receiving the benefits. Indeed, Gostin and Wiley (2016) explain that answers to questions such as “who” should regulate and what best serves the common good are characteristics that makes public health a “highly political” arena.

Sport-related concussion has also been framed as a public health concern (McNamee and Partridge, 2013; Harvey, 2014; Lowrey and Morain, 2014; Bachynski, 2019a). Goldberg (2008), an early proponent of ethical questions and public health dimensions of sport-related concussion, argues that sport-related concussion is a legitimate public health concern, questioning the role of private actors—in that case, the National Football League—who controlled the organisation and delivery of the health-care model to sports participants.

Bachynski (2019a) traces the evolution of the sport-related concussion “crisis” as a public health concern driven by advocates calling for a broader public health approach. Consistent with the earlier discussion on the importance of issue diagnosis and problem identification as foundations for the regulatory lifecycle, Bachynski, citing Gusfield, argues that “determining which individuals or institutions are given responsibility for addressing a problem is central to understanding how an issue is constructed as a public problem” (p. 201).

Entrenched social and cultural beliefs mean that the recalibration of this regulatory space has been jealously guarded and protected as a sports medicine issue, often met with resistance and in some cases, “emphatic condemnation” for views suggesting that other actors from outside sport have legitimate authority over the problem (Bachynski, 2019b, p. 197).

As a wicked regulatory problem, it is not surprising therefore that sport-related concussion has both private and public dimensions. This paper now turns to examine the governance framework through which the private dimensions of sport-related concussion are regulated by the NSOs within their sports.

## Governance Characteristics: a Comparative Review

As NSOs, the AFL and HC are regarded as “the cornerstones of sport governance” (MacDonald and Ramsay, 2016, p. 55). These NSOs in Australia and Canada are primarily responsible for the regulation of concussion in their sport in those countries. This is a function of their respective governance structures, which we now consider.

### The AFL—A Dual Governance Model

The AFL is the single regulatory authority for rule-making in the sport, although it embodies a dual model of governance. In the dual governance structure it is both organiser of the elite level professional club-based national league (league governance) and the recognised entity for the overall promotion and development of the sport (sport governance). This duality of roles derives primarily from the evolution and development of the sport. In recognition of this dual role, the AFL notes “Our obvious responsibility is to the national competition, but the AFL accepts responsibility for the development to the game, feeder structures and the overall health of the code” (Australian Football League, 1997, p. 15).

#### AFL League Governance

The AFL has its roots in the Victorian Football League, established in 1896 as the entity responsible for league governance of the original eight Victorian-based football clubs. In 1984, the VFL Board of Directors voted to appoint an independent Commission to administer the VFL competition which at the time had eleven based in Victoria and one in New South Wales (Independent Sports Panel, 2009). In the 1990s, the VFL changed its name to the Australian Football League following the expansion of the competition to other States in Australia.

The AFL Commission is responsible for the establishment of the national competition and the introduction of many strategies which have strengthened the elite level national competition (Crawford, 1993; Australian Football League, 1997). In terms of league governance, the AFL Commission considers itself “both keeper of the code and manager of the national competition” (Australian Football League, 1997, p. 15).

Following governance reforms recommended in 1993, the league governance model transitioned to incorporate the AFL Commission as an independent board of directors (Crawford, 1993). The AFL Commission is an independent decision-making body comprised of 10 elected members. Membership and voting rights in the AFL are vested in designated appointees as representatives from the clubs in the professional elite level national competition. The corporate constitution of the AFL defines the voting rights of its members and the power and duties of the AFL Commission and members (Australian Football League, 2018, cl 15 voting rights; cl 85 and 86 power and duties of Commission; MacDonald and Ramsay, 2016).

At the elite level of the competition, professional players are employed by the professional clubs that hold rights to participate in the elite level national competition. While the AFL is not considered the direct employer of the players, it asserts significant league governance control over players through its rule-making authority, the collectively bargained arrangements, and as a contractual party under triparte player contracts (Australian Football League and Australian Football League Players' Association, 2018). These labour market characteristics bring into focus the professional athlete as a particular type of worker (Brayton et al., 2019), and raises broader considerations of labour politics and production costs as elements to be considered in asserting regulatory legitimacy in league governance.

#### The AFL as an NSO

The National Australian Football Council (NAFC) was the original national governing body for the sport from 1906. Originally, members of the Council were delegates appointed by the various leagues which controlled the sport in their States and Territories. The NAFC was replaced by the Australian Football Foundation (AFF) to “administer football nationally on behalf of the AFL” (Australian Football League, 1995, p. 1). The AFF was recognised as having “close relationships” with the State bodies and consequently licence agreements were entered into for the “implementation of football development programs based on uniform national guidelines” the AFF later invested $5.2 million (AUD) in the sport's national development plan (Australian Football League, 1995, p. 1; Australian Football League, 1996, p. 32).

Governance changes were subsequently made, devolving power to the AFL Commission. According to MacDonald and Ramsay, the AFL “formally assumed national governing body responsibilities in 1995” and recognised as the NSO for Australian football (MacDonald and Ramsay, 2016, p. 94). Under the AFL Constitution, one of its objectives is to promote and encourage football and football matches both within Australia and elsewhere [2018 Constitution cl. 4 (b) (c)].

As the recognised NSO for the sport, the AFL has received significant financial benefits and “green light” regulatory exemptions from the federal government over the years (Pomfret and Wilson, 2011; Sport Canada, 2015). In return, the AFL as NSO is required to adhere to the mission-orientation of producing and delivering the sport across all levels of participation.

#### Australian Football—Governance Through a Network

The AFL governs the sport through a network of non-voting affiliates under affiliation or licence arrangements. State and Territory bodies affiliated with the AFL have authority to administer the sport at their respective levels in Australia. These entities are the AFL NSW/ACT (for the state of New South Wales and the Australian Capital Territory), AFL (Northern Territory), AFLQ for the AFL Queensland, South Australian National Football League, AFL (TAS), AFL (Victoria), and West Australian Football Commission Inc (WAFC). These SSOs are not members of the AFL and have no voting power under the AFL's constitution. Instead, the SSOs are affiliated by agreement with the AFL to administer the sport within their geographical boundaries. These affiliation agreements authorise the affiliates to “promote, develop, manage and control Australian Football” in their state or territory (West Australian Football Commission Inc. Constitution, 1).

These affiliates are each separate legal entities. In Western Australia, the WAFC acts as “caretaker” of football throughout the State but also owns two elite level clubs based in the State. This means that in the case of Western Australia, the WAFC is both an affiliate and club owner, operating through a not-for-profit model. Arguably, this enables the WAFC to have greater governance participation in the AFL at least over league governance considerations through its voting power as a member.

While each affiliate is recognised as a “Controlling Body” according to AFL Laws, the AFL regards itself as the “keeper of the code” and aims to “actively support” other levels (Australian Football League, 1997). For example, the AFL released the “Next Generation Australian Football Match Policy” aimed at players 5–18 years in 2009. The Next Generation Policy was developed by the AFL in collaboration with the affiliates and the Australian Sports Commission as it was then known. The Policy was revised in 2012 and renamed the “Junior Football Match Guide” (for the conduct of Australian football for junior players aged 5–12 years). The Guide is undated, but it appears from the Introduction that it followed a review in 2012. It is difficult to find any publicly available evidence as to whether the AFL has played any further role in terms of implementation or enforcement of these policies at the junior level of the sport.

#### Rule-Making Dominance of the AFL

In regulating the sport, the AFL has autonomy over its rule-making and standard-setting functions. It is not constrained by any international affiliations or global sports governance network. In accordance with the AFL's constitution, its rule-making function is one which falls for a vote; where members of the AFL, namely the Appointees designated by the professional clubs in the elite national competition, are empowered to vote at Annual General Meetings where substantial changes are proposed.

The AFL's exclusive power is reflected in the affiliation agreements with the State and Territory bodies, recognising the AFL as the national governing body for Australian Football (AFLQ Application for Affiliation, undated). To illustrate, the AFL's 2013 Member Protection Policy states that “the AFL retains the exclusive power to amend and vary the *Laws of Australian Football* from time to time” (Australian Football League, 2013, p. 5). The AFLs' exclusive power over rule-making is recognition of its regulatory role in steering and influencing behaviour in the sport.

The 2020 Laws of Australian Football (“AFL Laws”) apply to all matches of Australian Football as organised and conducted by a “Controlling Body” (Australian Football League, 2020a, p. 10). A Controlling Body includes the AFL and the AFL Affiliates (Australian Football League, 2020a). The AFL's exclusive power and rule-making authority is recognised by the AFL Affiliates. For example, the AFLQ recognises the regulatory authority of the AFL by noting that the AFLQ and all clubs licenced to or leagues affiliated with AFLQ are required to “adhere to all relevant rules, regulations, and policies of the AFL…” (AFL Queensland, 2020, clauses 1.5).

The AFL retains authority to sanction non-compliance with the rules at the elite national competition, but delegates authority to the AFL Affiliates to sanction non-compliance within their State and Territory levels (Australian Football League, 2020a, clause 22.4, 84). In that regard, the AFL Affiliates are involved in the implementation and enforcement of the rules. They are not, however, involved in the rulemaking as this belongs exclusively to the AFL and its voting members.

### Hockey Canada—A Federated Model

HC is the recognised NSO for the sport of hockey in Canada. It is a member of the International Ice Hockey Federation (IIHF) with responsibility for the governance and administration of the sport in Canada and representation of the country in international competition (HC Bylaws, Regulations, History May 2019). It has its roots in the Canadian Amateur Hockey Association (established 1914). The CAHA was formed to oversee amateur hockey at the national level in the country. It started with 21 delegates representing local and regional leagues across the country. Other leagues continued to join over the next 60 years, in large part to be able to vie for the national championship.

The Canadian Hockey Association (CHA) was established by the federal government in 1968 specifically to oversee the national teams. The CAHA continued to exist and divided into two tiers based on competitive level. In 1975, the Tier I teams established the independent Canadian Major Junior Hockey League (CMJHL; now the Canadian Hockey League) and connected with the professional level National Hockey League (NHL), which had been in existence since 1917 (emerging from the original National Hockey Association formed in 1909). The CMJHL separated completely from the CAHA in 1980, retaining only a loose affiliation with the NSO which is now based only on its releasing players for national team participation in the World Junior Championships.

In 1994 the CAHA and CHA merged to form HC, the official governing body with responsibility for the regulation and promotion of amateur hockey in Canada on a nation-wide basis, and management of national teams to participate in international competitions. Its mission is to “Lead, Develop and Promote Positive Hockey Experiences.”

Today, HC has one class of Members, who are the 13 constituted amateur hockey federations representing each province and territory (“the Members”). The Members elect (and can remove) Board of Directors. They make proposals to, amend or repeal any articles, bylaws, regulations or playing rules of HC. The elected Board of Directors comprises individuals with no formal affiliation with any of the Member federations. Their duty is to regularly review HCs terms of reference and approve all partner agreements.

#### Ice Hockey—Governance Through a Network

In turn, the 13 provincial/regional/territorial federations are responsible for the governance and administration of amateur hockey within their jurisdictions, and for representing their constituents at HC meetings. They are empowered by HC to manage and foster hockey at the regional and grassroots levels. Each federation has several associational members that oversee minor level (community club) hockey. Within the federal model of sport governance, each level of HC operates hierarchically but semi-autonomously with regard to policy and practices governing hockey within their own jurisdiction. Each federation has in its by-laws something to the effect that the federation and its members, “[in] an unalterable provision… shall operate in a manner consistent with the by-laws, regulations and rules of Hockey Canada” (By-Laws of the Ontario Hockey Federation, Article 2 Status with Hockey Canada etc. 2020).

HC's only affiliation with the NHL professional league (and other professional leagues in North America) is for the release of players for Canada's participation in the World Championships and Olympic Games.

#### Rule-Making Dominance of HC

As NSO of the sport, HC has rule-making authority, subject to the constitutional voting rights of its Members as representatives of the 13 provincial/regional/territorial federations. Members have power to vote to accept or reject changes to the Bylaws, Regulations, and Playing Rules by Ordinary Resolution (HC Bylaws, Regulations, History May 2019).

The governance and constitutional voting rights of members within HC establishes the rule-making authority of HC as the NSO. Nonetheless, the 13 provincial/regional/territorial federations retain discretion over how they implement and enforce the HC ByLaws, Regulations and Rules.

## Sport-Related Concussion as a Regulatable Concern

Before examining the various approaches and responses to concussion, it is important to briefly explain the differences between the sports of Australian football and ice hockey. We acknowledge that each sport is substantially different to the other and justifies a nuanced approach to injury prevention. We do, however, consider that once a concussive injury is sustained, there are synergies that exist in regard to injury management and return to play or practise protocols, a fact that underpins the principles-based approach established by the international Concussion in Sport Group (CISG) return to play approach (McCrory et al., 2017). However, each sport has autonomy to self-determine the design, implementation, enforcement and evaluation of concussion strategies and the sport-specific nuanced approaches.

### Two Sports

Australian football is a full bodily contact sport played on a real or synthetic grass football field with 18 players in total from two teams. All players have access to the entire field, with goal posts at either end of the field. As a full bodily contact sport, players connect with others on the field, connect with the ground, and occasionally connect with the stationary goalposts. Consequently, the risks of contact are heightened by contact with other players congestion in taking the ball, which is kicked and run from end to end. Australian football has high incidence rates of concussion measured in 2019 as 6.5 per 1,000 player hours (Australian Football League, 2019). Regarded as an indigenous sport, Australian football is not formally affiliated with any international federation. It is not recognised as an Olympic sport.

Ice hockey has been described as a “sport unlike any other” (Goodman et al., 2001), where five players (plus a goalie) from each team move at high speed across ice that is surrounded by immovable “boards,” making contact with rigid hard surfaces and other players. Consequently, concussion incidence rates in ice hockey are regarded as one of the highest across contact sports (Goodman et al., 2001; McCrory et al., 2017). Ice hockey is recognised as a Winter Olympic sport.

### Early Concussion Concerns

We now turn to consider early concussion concerns in each country and how the AFL and HC have responded with efforts to steer and influence behaviour. We examine these efforts with regard to concussion prevention, concussion injury management, concussion education and concussion research.

#### Concussion Concerns—Australia

With rising concerns about the safety of some contact and collision sports throughout a particularly violent period in Australian sport (Vamplew, 1991), several early studies identified the need for further research and harm mitigation strategies of concussive and head injuries in Australian sport (Chapman, 1985). In 1994, the Australian Commonwealth Government's National Health and Medical Research Council (NHMRC) through the Football Head and Neck Injuries Committee examined the impact of concussion in four codes of football, including Australian football. While the state of scientific understanding was evolving, the Committee noted the “absence of good overall data on injuries in football” and identified the need for the sports to take proactive steps to mitigate the potential harm (National Health Medical Research Council, 1994, p. xv).

Australian Football was one of the four codes of football identified by the NHMRC Committee. The NHMRC Committee did not limit their concern to elite levels of the national competition. Instead, they were concerned about the high numbers of participants of all ages and levels of skill (National Health Medical Research Council, 1994). The AFL, while not specifically recognised as having made a formal submission to the inquiry, was represented through affiliations with early research pioneers in the field—Dr. Hugh Seward, Dr. David Maddocks, and Dr. Paul McCrory, the latter noted as Medical Officer, Australian Football League and member of the NHMRC Medical Consultant Panel.

The NHMRC Committee published a report and made several recommendations. Key recommendations included the establishment of a national registry and centralising of data collection to understand incidence and prevalence, and common guidelines for concussion management for adoption by all codes. The Committee also recommended that illegal play be sanctioned in efforts to signify the seriousness of the harm. For example, the NHMRC Committee recommended that consideration be given to an increase in penalties for illegal tackles and that this should cover “all levels of football” (National Health Medical Research Council, 1994, p. 100).

It is difficult to ascertain from publicly available records whether the NHMRC recommendations were actioned by the AFL. As earlier noted, the AFL at the time of the NHRMC report in 1994 was transitioning from league organiser to also become recognised as NSO for the sport. This new role would likely have required the AFL to take steps to implement rules across the sport, and through its affiliated network. The absence of publicly available data makes it difficult to verify what steps, if any, were taken across all levels of the sport.

Apart from the funding of the NHMRC Committee and subsequent report in 1994 for the next two decades, the Australian Commonwealth Government adopted a “hands off” regulatory approach to the management of sport-related concussion (Greenhow, 2018). The AFL, as one of the sports noted in the NHMRC Report, continued to voluntarily and autonomously set, direct and control the regulatory responses to the issue within its sport with very little government interest from federal or state agencies.

The AFL first noted concerns about concussive injuries at the elite level of the sport in its 2007 Annual Report. The Report stated that “several injuries were becoming less common, with concussion in particular taking a downturn” (Australian Football League, 2007, p. 60). However, in 2008 the AFL introduced the first iteration of concussion management guidelines when a “broad based policy to cover the management of concussion” was implemented (Australian Football League, 2008, 2009, p. 55; Lane, 2011). The 2008 concussion management guidelines were designed for use by the clubs in the national elite level competition. These guidelines were revised several times across the years in line with developments in medical management and scientific understandings.

In response to recent scientific findings of CTE found in three former AFL professional players—the youngest and most profound being discovered in a 38-year-old former professional AFL player—the AFL publicly stated that it has been researching the issue of concussion in Australian football since 1985 (Australian Football League, 2012, p. 1). From publicly available information examined in this study, it is difficult to accurately trace the early stages in the evolution and development of the AFL's concussion strategy following the 1994 NHMRC Report. The AFL appears to have been focussed primarily on its league governance role at the elite national competition level.

#### Concussion Concerns—Canada

Several studies trace the historical and cultural concerns associated with sport-related concussion in North America, albeit with a particular focus on football (Harrison, 2014; Bachynski, 2019a). Early incidents of reported head injuries in ice hockey occurred in Canada, and were instrumental to initial responses by the CAHA (Bachynski, 2020). In 1965 the CAHA introduced a mandate for players 18 years and younger to wear helmets in the sport. While this piece of equipment eventually became normalised, the anecdotal and confirmed incidence of head injuries continued in ice hockey, particularly as the game continued to evolve with faster, bigger, and stronger players on the ice.

Into the twenty first century, concerns continued to be raised by researchers about the potential harm associated with mismanaging concussive injuries in hockey (Goodman et al., 2001). Although Canadian NHL players had suffered severe and even fatal head injuries as early as 1933 (Irvin “Ace” Bailey) and then 1968 (Bill Masterson), the 2011 high-profile repeated concussive incidents of Canadian NHL player Sidney Crosby sharpened the focus across the sport on the associated dangers (McGannon et al., 2013). The “Crosby effect,” as it came to be known, increased nationwide public awareness of concussion across sport (Sport Canada, 2015). In 2012, a special task force of the FPTSC was formed and conducted an inventory of existing prevention tools of concussion and head injuries in sport across Canada.

Sport related concussion became a high priority area for the federal government under Justin Trudeau's administration, with Mandate Letters issued by the Prime Minister in 2015, 2017, and 2019 in support of raising awareness and to implement a pan-Canadian concussion strategy (Mandate Letters from the Prime Minister to Ministers on Expectations and Deliverables, 2015). In 2015, the FPTSC hosted the Workshop on Concussion Prevention and Management (Sport Canada, 2015). At that, delegates from Sport Canada, the Public Health Agency of Canada, and several provincial governments were joined by non-governmental representatives including HC. The NSO has since been directly involved as a consultant to various public and public-private efforts aimed at addressing sport related concussion in Canada. It serves on the Government of Canada's Active and Safe Injury Prevention Initiative and consulted directly on the establishment of the 2017 Canadian Concussion Guidelines.

### Concussion Prevention—Rule Changes to Mitigate Risks

In this section we consider the steps taken by the AFL and HC to prevent concussive injuries from occurring in the first place. We are interested in how rules have been modified and equipment mandated to mitigate the potential risk.

#### AFL Approach

Under the AFL's regulatory mandate, several rule changes have been established intending to reduce the risks associated with sport-related concussion. From 2000, the AFL introduced rule changes designed to reduce dangerous play. For example, rules to penalise bumping and forceful contact were introduced in 2007 together with greater policing of dangerous tackles. Since 2009, further rule changes have been introduced with a more concussion-specific approach. For example, rule changes to penalise any dangerous tackle is now a reportable offence triggering review by the AFL Match Review Tribunal (Australian Football League, 2020b). These rule changes are also reflected in the AFL Laws and Concussion Protocols as amended from time to time.

Since 2016, penalties and sanctions have been imposed on professional players who have breached the AFL rules regarding head knocks. In 2021, the AFL publicly stated that the fines would be reinvested into concussion-specific research initiatives (Maddocks, 2021).

With growing recognition of concussion in other codes of football, questions often arise as to why helmets are in place in sports such as American football, but not in Australian football, where offensive play potentially comes from 360 degrees. The AFL does not require the wearing of helmets. Minimum standards are now embedded within the Collective Bargaining Agreement requiring other equipment such as mouth guards (Australian Football League and Australian Football League Players' Association, 2018, Annexure C). For amateur levels, the AFL has published a position statement regarding mouthguards and helmets on the AFL Community website.

#### HC Approach

After the CAHA's mandatory helmet rule in 1965, HC instituted two rules with the formal and specific aim of regulating concussion. In 1985 it introduced a rule to deter and ultimately eliminate hitting from behind. A no hit to the head rule was instituted in 2002. Both were accompanied by recommendations for penalty assessment, although that was and continues to be at the discretion of each federation. In 2011, the Annual General Meeting of Hockey Canada implemented a new Head Contact Rule with “zero tolerance,” to reduce the number of head injuries suffered in the game. As per the federal model, the rule was made mandatory for each of HC's member federations at the provincial/territorial level, and in turn the federations' association members governing hockey at the minor level in communities across the country.

Subsequently, and controversially, in 2013 Hockey Canada implemented a rule to prohibit any kind of body checking (not just hitting from behind) in the Peewee age group (11- and 12-year-olds) and younger. Although there was much concern about the implications of this new rule for players learning how to give and take a body cheque effectively, this change resulted in a 70% reduction in the risk of concussion in minor hockey across Canada. The rule prompted several community-level associations to consider extending the rule to older age groups, although it was ultimately supported in only one city league.

Equipment modifications have also been mandated by HC, recognising the nuanced approach to injury prevention given the nature and extent of bodily contact in ice hockey. Canadian Safety Association-certified (CSA) helmets, full face caged protectors and mouthguards are required for all youth programs and recreational leagues (although optional for senior men, who may not have worn such equipment before), and for all goalkeepers (Hockey Canada, 2019). Again, this regulation is adopted by the provincial and territorial federations, and subsequently the minor level associations, with penalties for rule violations designed and administered by the respective federations.

During international tournaments, which are for “U18” aged players (min. 15 years) and older, HC must follow the regulations of the IIHF regarding playing rules and equipment requirements. These regulations are consistent across the two hockey bodies; for example, “clean” bodychecking is allowed and hits to the head are not (International Ice Hockey Federation, 2019).

### Concussion Injury Management—Injury Surveillance

A key aspect of the management of sport-related concussion is to understand incidence and prevalence rates. This is made possible through injury surveillance. Finch et al. (2013) and other epidemiologists identify the challenges associated with the patchy data available on the issue of sport-related concussion. A key source of accurate data may be the sport's governing body voluntarily collecting and disseminating such information. Decisions about what to share with the public has been entirely a matter for the sports to decide. Further, decisions about whether to coordinate efforts to collect data across all levels of the sport—from amateur to elite, or to focus on elite levels remains primarily an NSO decision.

#### AFL Injury Surveillance

The AFL has been collecting data since commencing the annual injury surveillance system in 1992 (NHMRC 66). The collection of injury surveillance data aligned with early concerns about the cost burden of sports injury in general and their identification as a key health priority focus in 1997 (Commonwealth, Australian Institute of Health and Welfare, 1997). The first AFL injury surveillance report listing concussion in sport as an injury was published in1993, examining data collected from the 1992 season (Seward et al., 1993).

The AFL's injury surveillance was initially funded by an Australian Sports Injury Research Grant (National Health Medical Research Council, 1994). From 1995, the AFL has funded the surveillance itself and has unilaterally determined how it collects, analyses, and disseminates results. Most of the injury surveillance data is collected at the elite professional level in the national competition (Ryan, 2017). The data is then used to guide decision making about injury prevention, including the modification of rules and policies at the elite level through the involvement of the AFL Medical Officers Association (Australian Football League, 2019).

At the amateur levels of the sport, injury surveillance and reporting is sporadic, with most research coming from hospital admission data, insurance records when available and the efforts of researchers with a focus on injury prevention and control (Finch et al., 2013).

#### HC Injury Surveillance

At the time of writing, sport-based surveillance of concussion in Canada is in the early stages. Data on incidence rates by sport to date is taken from medical records within each province. The data are subject to variation in collection methods, including definition of concussion and recording of its mechanism (i.e., in sport and which sport). Any injury surveillance is ultimately dependent on self- or medical reporting, and is made more complex (and therefore less reliable) because of the difficulty diagnosing, and the reluctance in reporting (Finch et al., 2013). That will no doubt continue to be a factor as HC, along with all other Canadian NSOs, is mandated by the FPTSC to begin monitoring concussion in its sport by 2022.

### Concussion Injury Management—Guidelines

#### The AFL Approach

The AFL has implemented several initiatives at the elite level of the national competition in concussion injury management. In 2008, Concussion Guidelines were developed for injury management. These guidelines are periodically updated and outline common symptoms and injury management steps when a player is suspected of sustaining a concussive injury. In 2015, the AFL developed a AFL Match Head Trauma Assessment form, a sideline diagnostic tool for use by the Club Medical Officer to assist with diagnosis. Concussion injury management procedures are incorporated in the AFL Regulations [AFL Regulations 12.5, 12.8 (c) (ix)]. The latest version of the AFL and AFL Player Association Collective Bargaining Agreement now requires each club to have at least one doctor trained in the diagnosis and management of concussion in accordance with the AFL Concussion Guidelines (Australian Football League and Australian Football League Players' Association, 2018, p. 109; Tworney, 2021).

A video audit tool known as “Hawkeye” was introduced by the AFL in 2015 to enable medical staff to replay incidents from multiple angles and access complete information about head knocks to players as part of the match-day concussion management process (Clifton et al., 2017).

The AFL now monitors and enforces compliance with the concussion management procedures at the elite level of the national competition (Gleeson, 2021; Tworney, 2021). Clubs have been fined for failing to comply with these protocols. To illustrate, the Carlton Football Club was fined $20,000 in 2018 for breaching the concussion guidelines (Collins, 2018).

At the amateur level, the AFL provides some information to assist with concussion injury management (Australian Football League, 2021b). A concussion app was developed and released through collaboration with the Murdoch Children's Research Institute designed to assist parents and others to recognise when medical attention is required (Australian Football League, 2017; Murdoch Children's Research Institute, 2018). Through the AFL Community Club website, the AFL makes available links to Concussion Management Guidelines for use at amateur levels of the sport (http://www.aflcommunityclub.com.au)^1^. A “Community Football Head Injury Assessment” and Pocket Concussion Recognition Tool (CRT) is also available for download for community club use. It appears that the AFL does not play a direct role in terms of monitoring and enforcing compliance at the other levels of the sport. In some States and Territories, the Affiliate websites link back to the AFL Community Club website.

#### HC Approach

HC's Concussion Policy (Hockey Canada, 2018) acknowledges the importance of injury prevention and the education of all participants about recognising symptoms, however the Policy focuses predominantly on concussion management. A process for managing suspected concussion includes assessment using HC's own Hockey Canada Concussion Card—a two-page document that outlines common symptoms and signs of a concussion, key steps in immediate management, and a return to play strategy—or the SCAT 5 Pocket Recognition Tool. The injury management process, which aligns directly with the Canadian Concussion Guidelines that HC helped to establish, directs the player to be removed from play immediately with no return to play that day. Written clearance by a physician, along with a Concussion Followup and Communication Form, is required to return to play. Importantly, the Policy applies to all registered participants, coaches, officials, trainers, safety personnel, parents/guardians, administrators, and decision makers of Hockey Canada, with adherence expected. Given the hierarchical membership of the sport in Canada, this involves all parties affiliated with the Hockey Canada system. Nonetheless, each federation is empowered to determine the appropriate disciplinary action for any club, team, or individual who violates any aspect of the Policy.

In another example of the semi-autonomous nature of HC's federal model of governance, the Ontario Hockey Federation (OHF) is legally required to also adhere to the guidelines set out by “Rowan's Law,” which is legislation enacted in that province in 2018 [*Rowan's Law (Concussion Safety) Act*]^2^. The tragic death of 17-year-old rugby player Rowan Stringer in 2013 of Second Impact Syndrome (catastrophic swelling of the brain caused by an injury that occurs before a previous injury has healed), and the mismanagement of her earlier concussive injuries, signified that the issue was not just a professional sport concern and ultimately prompted legislation aimed at ensuring education and practise in support of concussion prevention and effective management. The OHF must adhere to Rowan's Law and thus has an Acknowledgment Form with links to age-appropriate resources that all federation members must address, along with an OHF Concussion Code of Conduct.

A related policy, but not specific to concussion, is HC's Safety Person Program. This initiative requires that each team has one designated individual present at any hockey practise or game who is responsible for engaging HC's Emergency Action Plan if injury occurs (or specific concussion management plan if head injury is suspected). A Safety Person trained through a HC module is a requirement for team participation. Again, one federation (OHF) offers what HC considers an equivalent program and so its constituent clubs and teams are exempt from the HC policy.

### Concussion Education and Advocacy

Knowledge dissemination is regarded as a critical part in developing an effective concussion strategy (Finch et al., 2013; McCrory et al., 2017). In steering and influencing behaviour, NSOs play critical roles in designing and implementing education and advocacy initiatives and using their position to change behaviour.

#### The AFL

The AFLs education agenda is primarily focussed on the elite level of the sport. Since 2013, the AFL has organised two conferences on concussion, the most recent in 2017. The conference topics were relevant to concussion issues at the elite level of the sport, with guest presentations the Chief Medical Officer from the National Football League (Australian Football League, 2017). One presentation, however, reviewed the management of concussion in children.

The AFL Community webpage provides a link to the 2017 AFL Concussion in Football Symposium. This website also provides links to some concussion resources. It is difficult to find publicly available evidence of other initiatives aimed at concussion education at the amateur level of the sport^3^.

In 2019, the Australian Institute of Sport and partners released the third version of the Concussion in Sport position statement as an initiative co-ordinated by the Australian Commonwealth Government. This statement includes information to educate parents and other stakeholders at the amateur level of sport. The AFL is noted as a supporting organisation and therefore supports the general principles of the Concussion in Sport Australia position statement' (www.concussioninsport.gov.au).

#### Hockey Canada

As part of its involvement in the FPTSC Concussion Group and through the partnership with Parachute, a pan-Canadian injury prevention non-profit, and other peak industry bodies, HC has been an active participant in developing resources and educating stakeholders across sport in Canada. It has also committed to education about concussion in hockey through its Concussion Policy and sport-specific resources. Although HC's Concussion Policy focused on concussion management, it does “encourage the prevention of concussions using sound education programs” and refers to “education of all participants on the prevention and recognition of head injuries and responsible return to play” (Hockey Canada, 2018). Again, federation members, and their constituents in turn, are bound by the Policy and so are made aware of its educational component.

Importantly, HC has a webpage dedicated to concussion resources. It contains a wealth of information, including “Facts and Prevention,” “Sport Concussion Assessment,” and HC's own “Concussion Toolbox.” The concussion resources page is, however, embedded within the general “Safety” page of the “Hockey Programs” page that is linked at the bottom (rather than on a banner) of HC's homepage. It has no more prominence than information about general Hockey Canada Safety Programs, the general Emergency Action Plan for any hockey injury, and the anti-bullying and harassment Respect in Sport program.

The website also links to injury prevention charity Parachute Canada's Canadian Guideline on Concussion in Sport, which was developed with consultation with key stakeholders including Hockey Canada. These guidelines form a central part of Canada's Concussion Protocol Harmonisation Project, which aims to develop core principles and achieve consistency across the entire Canadian sport community. HC, like other Canadian NSOs, benefits by having access to “evidence-based concussion protocols that were harmonised with the Canadian Guideline…yet tailored to meet the specific needs of each sport” (Parachute Canada)^4^.

### Concussion Research

Both the AFL and HC have participated in several international concussion symposia, commencing in 2001 with the inaugural First International Symposium on Concussion in Sport held in Vienna. The importance of establishing research strategies and priorities is now regarded as a critical part of concussion in sport. The quest to understand the aetiology and epidemiology of this sports phenomenon has resulted in extensive research drawn both from within sport and external to the sport.

#### AFL Research Initiatives

In 1999, the AFL Commission appointed the AFL Research Board to administer the selection of research priorities and allocation of funding (AFL Research Board Policy and Guidelines, on file) (Australian Football League, 2011). Several projects have received Research Board funding to advance scientific understanding of concussion (Hanlon, 2010). The existence of the AFL Research Board is a testament to its commitment to directly supporting the generation of new knowledge about concussion in its sport. In the latest CBA with the AFL Players Association, for example, the AFL has agreed to commit $250,000 per annum toward concussion research, collected from tribunal fines with shortfall to be paid by the AFL (Australian Football League and Australian Football League Players' Association, 2018, clause 43).

The AFL has reported that it has invested significant funds toward scientific research into understanding the impact of concussion in its sport and concussion has been on the agenda of the AFL for decades (Thompson, 2014). In 2008, the AFL Research Board funded a project that led to the development of the Concussion Guidelines (Australian Football League, 2008).

In 2013, the AFL partnered with the Florey Institute of Neuroscience and Mental Health through its connexion with Paul McCrory (Florey). This partnership has provided the AFL with access to neuroimaging diagnostic tools such as function MRI technology. Several AFL players have been tested as part of research projects to understand the functional changes as result of concussive injuries.

Since 2001, several AFL representatives have presented at the CISG Concussion in Sport conferences. The AFL has close affiliations with several members of the CISG group and presented the findings of several AFL-funded research projects at the 5th International Concussion Symposium in Berlin in 2016 (Clifton, 2017). At the amateur level of the sport, the AFL Research Board engages with the AFL Affiliates to ascertain research priority areas (AFL Research Board Policy, clause 7).

#### Hockey Canada Research Initiatives

In contrast, HC has had little direct financial engagement in concussion research, focusing its efforts instead on education and advocacy. Nonetheless, it has had a voice in the CISG, of which Dr. Mark Aubry, Chief Medical Officer for Hockey Canada, is a founding member and co-author of the Consensus Statement on Concussion in Sport (McCrory et al., 2017).

## Discussion—Two Sports, Two Systems, One Goal

The AFL and HC are two sports, operating under two different systems, with one goal in common—to direct and influence behaviour around sport-related concussion. Consistent with sociological (new) institutionalism, both NSOs have and assert regulatory legitimacy founded in the “valid, objective social feature[s]” of their governance structures, that is further strengthened by their broader cultural legitimacy as the “social object [that is] right” (Batuev and Robinson, 2019, p. 169) in their respective countries. Both aspects are supported by their historical and cultural roots as the peak bodies for their sport, as evidenced by their long and sustained histories with the evolving governance of Australian football, and ice hockey in Canada. The predominantly normative instruments—concussion policies, protocols, surveillance systems—that frame the AFL's and HC's regulation of this injury reflect, and have been influenced by entrenched cultural values.

Yet the regulatory legitimacy of the AFL and HC—the credibility and acceptability as the rule-making authority— may be all the two NSOs have in common. At a fundamental level, they each have different governance models that align with their historical and cultural roots. The AFL, with the dual model of governance, and HC with the federated model, operate under very different rule-making systems that have implications for establishing the regulatory agenda regarding concussion. The AFL's responsibility for league governance attracts a greater proportion of its attention, certainly as far as regulating concussion and the role of clubs as voting members of the AFL captures the AFL somewhat in terms of regulatory oversight. These governance characteristics determine who participates in setting and directing the regulatory agenda and, indeed, the priority or otherwise of what is classified as a regulatable concern. HC involves direct member participation through constitutional voting rights, whereas the AFL Affiliates do not. Consequently, prioritising concussion as a regulatable concern is likely to have been more closely aligned to voting member interests in HC and, until very recently, a more marginal issue for AFL sport governance as a reflection of voting interests within the AFL.

Importantly, the NSOs also have different regulatory target audiences in mind; where the AFL remains primarily focused at the elite level while HC takes a broader “whole of sport” approach. Consequently, they approach concussion prevention differently. The AFL is focused on elite level rules, compliance and enforcement, while HC is focused on all of sport rules and equipment, albeit with a particular focus on adolescents. They approach concussion research differently, with the AFL's financial investment in research and HC providing more in kind support through expert opinion. They also approach injury surveillance differently—where the AFL has an established injury surveillance system focused at the elite level while HC does not currently monitor concussion in hockey.

When addressing their regulatory agendas for concussion management, the NSOs' collaborations and networks also differ. HC partners closely with public and other private agencies; while the AFL tends to operate autonomously. Consequently, this influences the broader sport-related concussion discussion in their country with HC directly involved in and shaping that conversation, while the AFL is involved more indirectly.

Arguably, HC has played a much more active and direct role in collaborating with external stakeholders including state-actors, injury prevention charities and other sports. The HC cross-sport collaboration indicates a “whole of sport” approach, rather than the sharing of medical knowledge by chief medical officers as has been the case in the AFL.

For the most part, the AFL has set, directed and controlled how it regulates concussion in the sport. Most of its efforts have been directed at the elite national competition level. Further, while there has been some collaboration with external stakeholders, the AFL has primarily focused within its sport.

As noted by Hall (2018), “Canada is quickly becoming a leader in concussion prevention and management” and HC is an influential partner in that effort through its continuing contribution to the joint public and private establishment of a science-based guidelines and framework for concussion management in sport. While some have questioned this view (Mick, 2012), it does demonstrate the different regulatory agendas across the two sports in regard to sport-related concussion.

## Actionable Recommendations

Based on the above observations, we make two recommendations in regard to NSOs' policy-making and practices around sport-related concussion. The first recommendation is for NSOs to embrace their regulatory legitimacy—however designated and earned—in establishing and enacting a regulatory agenda with meaningful direction and influence over behaviour. Adopting Black's (2002) conceptualisation of regulation, NSOs can be engaged in the sustained and focused attempt to alter the behaviour of others according to defined standards and purposes with the intention of producing a broadly identified outcome or outcomes.

To illustrate this recommendation in the context of the AFL, we argue that it has the capacity and, importantly, regulatory legitimacy, to support greater engagement in support of stakeholders at the state and amateur levels of the sport. The Affiliates already acknowledge the rule-making authority and standard setting role of the AFL as the NSO. It is plausible, therefore, that the Affiliates already recognise the AFLs regulatory legitimacy over sport-related concussion.

The AFL appears to have been constrained by the historical and cultural governance structures that give voting rights to the clubs operating at the elite level in the national competition, creating a form of capture in decision-making. Consequently, the AFL, while carrying dual governance responsibilities, has exercised its policy-making function by addressing concussion with a primary focus at the elite level. Perhaps this is unsurprising when considered in the context of the historical and cultural influences in the sport. However, we argue that by adopting a “whole of sport” approach to concussion regulation, there is potential to yield greater dividends by enhancing trust that playing the sport of Australian football, at any level, is optimally safe for participants.

The second recommendation is for NSOs to build their broader cultural legitimacy through leadership by engaging in important public and private issues. As guardians of their sport, NSOs have capacity to build their cultural legitimacy which in turn underpins and enhances their regulatory legitimacy.

To illustrate, there is strong evidence in support of the advocacy role of NSOs in raising awareness and mobilising resources to highlight concussion as a public health concern, particularly in youth sport. HC's participation in the FPTSC Concussion group and subsequent involvement in partnerships with non-profit groups and government agencies fostered greater cultural legitimacy across the sport. The AFL, on the other hand, has remained true to its historical and cultural league governance roots, without venturing beyond in any substantial way, to participate in coordinated public advocacy of sport-related concussion.

In promoting the public health significance of sport-related concussion, there is much to be gained by stepping outside formal regulatory structures, creating opportunities to restore or enhance trust and cultural legitimacy. For example, when tracing the historical and cultural development of concussion in youth football in the United States, Bachynski (2019a) explains that the NFL, albeit after significant pressure from multiple sources, became an advocate for change and “actively participated in framing concerns about youth football head injuries as an issue to be addressed by better medical and coaching supervision” (p. 205). The NFL has no regulatory legitimacy over the amateur stream of American football and, indeed, over youth sports in general, but used its platform to engage in public health campaigns targeting the most vulnerable in the sports system.

## Conclusion

The above analysis establishes the various ways in which the AFL and HC as NSOs have asserted regulatory legitimacy over the field. Our research has identified the similarities, differences, and nuances in the way the AFL and HC, as institutional actors, govern through regulation to manage and minimise the harm associated with concussion. This comparative study has demonstrated that despite cultural and governance differences, the AFL and HC are both institutional actors asserting their regulatory authority by steering and influencing behaviour to manage and mitigate the harm associated with concussion in their sport.

This research has not examined how these actors monitor compliance or enforcement of their concussion policies, regulations and guidelines. Nor does this study assess whether SSOs or affiliates accept the regulatory legitimacy of the NSOs. Future research in this area could proffer insights into gauging views of stakeholders as to the credibility and acceptability of the NSOs policy-making authority, and other key components of the regulatory policy process. Primary data collection, such as through interviews, could build on the foundational assessment, and comparison, provided here. Further, a larger scale comparative study could include an assessment of compliance motivations, enforcement mechanisms and evaluation methodologies to determine the “fitness for purpose” as evaluation goals.

Despite the absence of a formal legislative mandate, both the AFL and HC assert regulatory legitimacy aligning with sociological institutionalism. They are institutional actors with strong historical and cultural roots in their sports. Their governance structures, albeit different in nature and form, provide them with the framework through which to exercise their rule making authority. These two sports, deriving from two systems, have the one common goal—to manage and mitigate the risk associated with the harm of concussion in their sport.

## Data Availability Statement

The original contributions presented in the study are included in the article/supplementary material, further inquiries can be directed to the corresponding author/s.

## Author Contributions

All authors listed have made a substantial, direct and intellectual contribution to the work, and approved it for publication.

## Conflict of Interest

The authors declare that the research was conducted in the absence of any commercial or financial relationships that could be construed as a potential conflict of interest.

## Footnotes

^1^In April 2021, the AFL announced new guidelines for concussion management in community sport signally greater interest across these levels of the sport.

^2^Rowan's Law (Concussion Safety) Act, S.O. 2018, c 1, ss 1-7.

^3^Recent additions to the AFL Education website now provide more concussion information for other levels of the sport. (Australian Football League, 2021a).

^4^Parachute Canada. Available online at: https://parachute.ca/en/injury-topic/concussion
